# Endovascular therapy of direct dural carotid cavernous fistulas – A therapy assessment study including long-term follow-up patient interviews

**DOI:** 10.1371/journal.pone.0223488

**Published:** 2019-10-17

**Authors:** Lorenz Ertl, Hartmut Brückmann, Maximilian Patzig, Gunther Fesl

**Affiliations:** 1 Institute of Neuroradiology, University Hospital, LMU Munich, Munich, Germany; 2 Radiologie Augsburg-Friedberg ÜBAG, Augsburg, Germany; Universitatsklinikum Freiburg, GERMANY

## Abstract

**Purpose:**

Endovascular embolization nowadays is a well-established treatment option for direct carotid cavernous fistulas (dCCF, Barrow Type A). There are many publications on the complication and success rates of this method. However, little is known on the patients´ opinion on the treatment result after several years. We report on this issue also including the “pioneer patients” treated almost two decades ago.

**Methods:**

We retrospectively reviewed the records of all patient (n = 25) with a more than 24 months follow-up interval after endovascular treatment of a dCCF at our institution from 01/1999 to 08/2018. We determined primary therapy success, complication rate, state of the fistula in the last imaging follow-up and quoted the patient’s subjective perception of the long-term treatment success using a standardized interview form.

**Results:**

Occlusion rate in the last imaging follow up was 96% (24/25) with a complication rate of 8% (2/25). The response rate on our interview request was 96% (24/25) with a rate of considered feedback of 84% (21/25 patients). Duration of our observation interval for the patient reported outcome was 143 months / 11 years (median, range: 35–226 m / 2–18 y). Most of them (21/25, 84%) felt they benefited from the treatment.

**Conclusions:**

Endovascular supply of dCCF is a highly effective treatment method leading to a sustainable therapy success with long-lasting stable subjective benefit even to our “pioneer patients” treated almost two decades ago.

## Introduction

Carotid cavernous sinus fistulas (CCF) are acquired arteriovenous (AV) shunt communications between the carotid arteries and the cavernous sinus. They are commonly classified into four types (A-D) according to Barrow. [1] A Type A CCF is characterized by a direct high-flow shunt between the internal carotid artery (ICA) and the cavernous sinus. Such direct CCFs (dCCF) are usually caused by a head trauma, or–less frequently—due to rupture of an internal carotid artery (ICA) aneurysm within the cavernous sinus. [2]

Endovascular techniques have emerged to a well-established treatment option for dCCF and there are many publications on the complication and success rates of this method. [3] These mainly focus on technical details and angiographic results. Available follow-up analysis report on an observation interval of up to four years. [4–8]

However, little is known on the patients’ condition and their subjective perception of the treatment result several years after endovascular treatment and some authors recommend corresponding studies. [5,9] In this article we report on this issue.

## Material and methods

### Study population

We reviewed the PACS, the electronic database and the paper based medical documentation of our hospital to identify all patients who received endovascular treatment of a CCF Barrow Type A (dCCF) at the neuroradiology department of our institution from 01/1999 to 08/2018. From these we included all cases with a minimum of 24 months follow-up period (treated from 01/1999 to 08/2016). Written informed consent to participate in this study was provided by all patients.

### Evaluation criteria

All patient records (paper based & electronic) were retrospectively reviewed to determine the clinical symptoms at the time of initial presentation and during the follow-up period. The imaging material was re-analyzed in detail by two experienced neuroradiologists (LE and GF, 8 and 16 years of experience respectively). The vascular treatment approach, materials used, and any complications related to the intervention with regards to vessel dissections, thromboembolic events, hemorrhages, infarctions, lasting disabilities and death were recorded.

To determine the primary success rate, the final angiographic result of the last endovascular session was assigned to one of four categories: (1) complete occlusion (CO), (2) downgrading (D), (3) unchanged (U), (4) upgrading (U, = increased AV-shunt volume).

Downgrading (D) was defined as a reduction of the AV-shunt volume in digital subtraction angiography compared to the state prior to the intervention. Residual AV-shunt volume had strictly to be confined to the sinus and must not involve the cortical veins (= no evidence for cortical venous drainage, CVD).

To determine the mid-term success all clinical and imaging follow-up examinations whatever of kind in the patient record were reviewed. The state of the fistula in the last available imaging follow-up was compared to the state prior to and immediately after treatment.

To evaluate the patient`s view on the treatment process a self-designed standardized questionnaire was sent to all patients. We asked them about their clinical symptoms on admission, at the time of discharge and at the time of the interview. Finally, a summary statement on the development of their fistula-related symptoms over time by choosing one out of three categories (“better”, “equal”, “worse”) was requested and a free-comment text field was provided. (S1 File)

We cross-checked the patients´ responses in the self-assessment form for any discrepancies to the findings documented in the medical records for the time points of admission and discharge.

There was no standardized clinical re-evaluation of the patients at the time of the interview.

### Interventions

All endovascular interventions were performed by trained neuroradiologists on a biplane neuroangiography suite (Neurostar Top or Axiom Artis zee biplane, both Siemens AG, Healthcare Sector, Erlangen, Germany).

### Data collection and statistical analysis

All data were collected in a custom-designed database using standard software (Access 2010; Microsoft, Redmond, WA, USA). All statistical analysis was performed using MS Excel 365 (Microsoft, Redmond, WA, USA).

The ethics committee of our institution approved this study (ID: 204–15, Ethikkommission bei der LMU München).

## Results

### Study population

We included 25 patients (14 female / 11 male) with a more than 24 months follow-up period after endovascular treatment of a dCCF at our institution (treated between 01/1999 and 08/2016). Median age at the time of treatment was 53 years (range: 22–78 y).

### Etiology & initial symptoms

Etiology of the dCCF was head injury in 19/25 (76%), spontaneous rupture of an intracavernous ICA aneurysm in 4/25 (16%) and iatrogenic complication after sphenoidal surgery in 2/25 (8%) of the cases. (Table 1)

**Table 1:** Comparison medical documentation vs. long-term follow-up interview.

ID	Etiology	Symptoms prior to the intervention as documented in the medical records					Symptoms prior to the intervention as quoted in the retrospective LFU interview		
		Chemosis, Exophthalmos Retroorbital pain, Ophthalmoplegia	Diminished visual acuity	Increased intraocular pressure	Pulsatile Tinnitus	Angiographically proven CVD	Chemosis, Exophthalmos Retroorbital pain, Ophthalmoplegia	Diminished visual acuity	Pulsatile Tinnitus
#1	Polytrauma after traffic accident, severe head injury with brain contusion, ICH and temporal bone fracture	+	-	-	+	+	+	-	+
#2	Head injury with brain contusion and multiple ICH following fall	-	-	-	-	+	-	-	-
#3	Traffic accident, polytrauma, head injury, skull fractures, facial & abducens nerve palsy, traumatic ICA-dissection, spine fractures	+	+	-	-	+	-	+	-
#4	Head injury, traumatic ICA rupture and dissection, extensive AV-shunt and CVD	+	+	-	+	+	+	+	-
#5	Ruptured intracavernous ICA giant aneurysm	+	+	+	+	-	-	-	+
#6	Head injury following bicycle accident	-	-	-	+	-	N/A	N/A	N/A
#7	Head injury following fall	-	+	+	+	-	-	+	+
#8	Head injury	+	+	+	+	-	+	+	+
#9	Traffic accident, polytrauma with head injury, cerebral edema & contusions, traumatic SAH, skull fratures, aortic rupture	-	-	-	+	+	-	-	+
#10	Iatrogenic ICA-perforation following paranasal sinus surgery	+	+	N/A	-	-	+	+	-
#11	Work accident with high free-fall drop, polytrauma with head injury	+	+	+	-	-	+	+	-
#12	Traffic accident with head injury, skull fractures	+	+	+	+	-	-	-	+
#13	Head injury following fall	+	+	-	+	-	-	-	+
#14	Spontaneously ruptured intracavernous ICA-aneurysm	+	+	-	-	-	N/A	N/A	N/A
#15	Polytrauma following brawl, head injury with skull fractures, thoracal contusion and abdominal blunt trauma	+	+	-	+	-	N/A	N/A	N/A
#16	Head injury following brawl	+	+	+	+	-	+	+	+
#17	Head injury	+	+	+	+	-	+	+	+
#18	Spontaneously ruptured intracavernous ICA-aneurysm	+	+	+	+	-	+	+	+
#19	Work accident, head injury following horse kick, traumatic SAH, EDH, skull fractures	+	+	+	N/A	+	N/A	N/A	N/A
#20	Iatrogenic ICA-Penetration following paranasal sinus surgery, unstoppable massive bleeding into the para- / nasal cavities, therapeutic ICA-occlusion	-	-	-	-	-	-	-	-
#21	Bicycle accident, head injury, multiple skull fractures	+	+	+	-	+	+	+	-
#22	Preexisting multiple sklerosis involving the optical nerve, traffic accident with polytrauma, multiple skull fractures, SDH, brain contusion and brain edema	+	+	+	N/A	-	+	+	+
#23	Spontaneously ruptured intracavernous ICA-aneurysm, Emergency intervention	+	+	N/A	-	-	+	-	-
#24	Head injury (° III) following fall, SDH/EDH/SAH, VP-shunt due to secondary malresorptive hydrocephalus	+	+	-	-	+	+	+	-
#25	Bicycle accident, head injury° III, SDH/EDH/SAH	+	+	-	-	-	+	+	-
		**20/25 (80%)**	**20/25 (80%)**	**11/25 (44%)**	**13/25 (52%)**	**8/25 (32%)**			

+ = symptomatic,— = not symptomatic, Abbreviations: LFU = long-term follow-up, ICH = intracranial haemorrhage, ICA = internal carotid artery, AV = arteriovenous, CVD = cortical venous drainage, SAH = subarachnoid haemorrhage, SDH = subdural haematoma, EDH = epidural haematoma, VP = ventriculoperitoneal

Based on the medical records chemosis, exophthalmos, retroorbital pain and / or ophthalmoplegia was present in 20/25 (80%) patients prior to the intervention. In 20/25 cases (80%) the visual acuity was diminished and 11/25 (44%) patients presented with an elevated intraocular pressure (IOP). In 13 out of 25 cases (52%) a pulsatile tinnitus was documented. Cortical venous drainage was angiographically proven in 8 out 25 cases (32%). There was a high level of correlation between the patient’s answers in the retrospective long-term follow-up interview and the documentation in the medical records. Answers matched to the information provided in the medical records in 54/62 (87%) of the available queried matched-pair items. (Table 1)

### Treatment details, complications & primary success rates

One patient (1/25, 4%) was treated by using only coils. 12/25 patients (48%) were treated with one or more detachable occlusion balloons (DOB). In an equally large number of cases (12/25, 48%) a combination of different materials as listed in Table 2 was used. (Table 2)

**Table 2:** Material.

Material		n	*Primary interventional occlusion*	*Primary interventional downgrading* *with secondary occlusion*	*Primary interventional downgrading* *with unchanged residual AV-shunt*
Coils only		1	1/1 (100%)	-	-
DOB only		12	11/12 (92%)	-	1/11 (8%) → patient # 16
Combined materials					
	DOB / Stent / RB	1	1/1 (100%)	-	-
	DOB / Stent / Coils	2	1/2 (50%)	1/2 (50%) → patient # 3	-
	DOB / Stent / Coils / RB	1	1/1 (100%)	-	-
	DOB / Coils / Onyx / DOB / RB	1	-	1/1 (100%) → patient # 19	-
	Coils / Stent	1	1/1 (100%)	-	-
	Coils / Covered Stent	1	-	1/1 (100%) → patient # 23	-
	Coils / RB	1	1/1 (100%)	-	-
	Coils / Covered Stent / RB	2	2/2 (100%)	-	-
	Coils / Stent / RB	1	1/1 (100%)	-	-
	Coils / Onyx / RB	1	1/1 (100%)	-	-
**Total**		25			

Abbreviations: DOB = Detachable occlusion balloon, RB = Remodelling balloon (temporary)

19/25 of the patients (76%) were treated via the arterial branch solely (TA), while in 6/25 cases (24%) treatment was performed by combining a transarterial with a transvenous approach (TA/TV).

14/25 patients were treated in one single endovascular procedure (56%), while in 8/25 cases (32%) two and in 3/25 cases (12%) three treatment sessions were needed. If multiple treatment was necessary, the median time interval between the first and the last session was 14 days (range: 2–49 days). All the patients in need of at least one more treatment session either remained symptomatic or showed early recurrence of their initial symptoms within this time interval. There was a fair high rate of early treatment failure among the patients in which a DOB was chosen as a first line option. In 8/13 patients (62%) in which a DOB was used in the first session, it showed secondary dislocation or pressure loss in the following leading to early fistula recurrence. Looking at it the other way round a DOB was used during the first intervention in 8/11 (73%) of all patients in need of several treatment sessions. (Table 3)

**Table 3:** Multiple treatment sessions.

ID	Etiology	Course	Time interval first to last treatment session (d)	Mid-term result
#3	Head injury	(1) DOB + Stent → fistula recurrence (2) TVC → Downgrading with minimal residual AV-shunt	6	CO
#4	Head injury	(1) TAC → fistula recurrence (2) TVC →complete occlusion	40	CO
#12	Head injury	(1) DOB → fistula recurrence (2) DOB → complete occlusion	2	CO
#16	Head injury	(1) DOB → fistula recurrence (2) DOB → complete occlusion	42	D
#17	Head injury	(1) DOB → Patient immediately developed ptosis, mydriasis and massive left sided headache → DOB removed → no permanent morbidity (2) Covered stent → fistula recurrence (2) Stent adjusted by remodelling balloon → complete occlusion	42	CO
#18	Ruptured ICA aneurysm	(1) DOB → fistula recurrence (2) DOB → complete occlusion	3	CO
#21	Head injury	(1) DOB → fistula recurrence (2) DOB → complete occlusion	8	CO
#22	Head injury	(1) DOB → fistula recurrence (2) DOB → fistula recurrence (2) DOB → complete occlusion	14	CO
#23	Ruptured ICA aneurysm	(1) Covered stend → stentangioplasty of a ruputured intracavernous ICA-aneurysm → downgrading (2) TVC → complete occlusion	2	CO
#24	Head injury	(1) Covered stent → downgrade (2) Covered stent → downgrade (2) TVC → complete occlusion	49	CO
#25	Head injury	(1) DOB → fistula recurrence (2) TVC → Complete occlusion	19	CO
			Median 14 (2–49)	

Abbreviations: DOB = detachable occlusion balloon, TVC = tranvenous coiling, AV = arteriovenous, TAC = transarterial coiling, ICA = internal carotid artery, FDS = Flow-diverter Stent, CO = Complete occlusion, D = Downgrade

Among 25 treated patients two complications occurred (8%). (Table 4)

**Table 4:** Complications.

PatID	Complication
#14	Thrombotic vessel occlusion (ICA) → Embolic ischemic stroke (left MCA) with secondary bleeding in the infarct territory → Patient finally died of recurrent coincidental cardio-embolic strokes seven years after successful fistula occlusion
#19	Dislocated DOB → Embolic ischemic stroke (left MCA)

Abbreviations: DOB = Detachable occlusion balloon, ICA = Internal carotid artery, MCA = Middle cerebral artery

In one case (pat #14, dCCF due to a ruptured ICA giant aneurysm) it came to a temporary thrombotic carotid vessel occlusion after deployment of a stent despite antiaggregation with heparin and tirofibane. It was successfully resolved by immediate i.a. fibrinolysis with 13mg TPA, however there remained a small embolic infarction in the posterior left insula with secondary bleeding in the infarct territory, not clinically apparent. The patient already showed preexisting cardio-embolic infarct scars and died seven years after successful fistula occlusion due to recurrent cardio-embolic strokes. (Table 4)

In one case (pat. #19) a DOB dislocated during an additional transarterial coiling in the same treatment session. Impact of this complication on the patient`s outcome is difficult to assess due to his extensive pre-existing injury resulting from a horse kick. The patient initially was admitted in intubated and ventilated state with trauma related subarachnoid hemorrhage and extensive CVD due to a traumatic dCCF. His unfavourable clinical course included surgical aortic root replacement, intracranial vasospasm, pneumonia, septic multiorgan failure and severe critical illness polyneuropathy. The patient remained in need of permanent care and died several months after the hospital discharge due to recurrent pulmonary infections. (Table 4)

Following endovascular treatment, the dCCF was completely occluded in 21/25 cases (75%), while in 4/25 cases (16%) a downgrading could be achieved. In none of the cases treatment led to an unchanged or deteriorated AV-shunt, thus improvement compared to the initial state was achieved in all of the cases.

### Mid-term follow-up

To assess the mid-term follow-up success rate fistula state in the last available imaging follow-up was determined. Any imaging modality (CT, MRI, DSA) was considered. Duration of the mid-term follow-up defined as the median time interval between treatment and date of the last follow-up imaging was 4 months (range: 0–202, median: 4, mean: 37.7). In one case (1/25, 4%) the primarily achieved downgrading situation remained unchanged, while 24/25 patients (96%) showed no evidence for AV-shunt in the last follow-up imaging examination. None of the patients presented with a secondary upgrade or recurrent dCCF. This means that in all cases with primary occlusion by intervention only the therapy results remained stable (21/25). In addition, however, 3/4 patients (# 3, 19 & 23) with downgrading after the initial treatment sowed a secondary spontaneous occlusion during the mid-term follow-up (75%), while just one patient (#16) showed a stable downgrading with a small residual AV-shunt volume equal to the primary result. (Table 5)

**Table 5:** Angiographic therapy results & long-term follow-up interview.

ID	Patient age by treatment	Primary result	Mid-term result	Patient age by interview	LFU interview	
					Summary statement	Free text comment
#1	60	CO	CO	69	Better	„After the injury I suffered from squinting and double images (staggered diagonally). After recurrent eye surgery, the strabism was corrected to some extent. Concerning the double-images many futile attempts were made with glasses cover, prism lens, etc. Currently I get along quite well with an occlusion lens on the left eye. The defective vision of the right eye is corrected with a sliding vision goggle, the left eye is constantly tearing. The deafness of my left ear was remedied by an operation in 2011, the residual hearing impairment is supplied with hearing aids. The left sided facial paresis still exists. Up to this day I sometimes have acoustic hallucinations, which were a psychological burden especially within the first years after the accident“
#2	66	CO	CO	82	Equal	„Since the accident in summer 2002 my wife is in need of permanent long-term care “
#3	22	D	CO	40	Better	„All deficits caused by an accident (severe brain trauma with damage to the right optic nerve and the right auditory nerve). Two weeks after the accident my fistula was occluded, another two weeks later the right eye was enucleated.“
		*→ Secondary occlusion →*				
#4	73	CO	CO	87	Better	"My wife will be 87 years old. She has a good memory, but must occasionally search for terms or names. About five times in the last two years she was somewhat confused after waking up. She has a macular degeneration in addition to her brain nerve palsy. She sees poorly and doubled and therefore can't drive anymore. Epilepsy 1x without cramping 6 months ago. All in all she's doing better than many others of her age. The operation saved her life. We would like to thank you for your excellent performance. How you have sealed the "leak" by coiling is a miracle! "
#5	53	CO	CO	59	Better	-
*#6*	*46*	CO	*CO*	*52*	*No response*	* -*
#7	44	CO	CO	62	Better	"The quality of my life has improved considerably. Thank you very much!"
#8	59	CO	CO	70	Better	-
#9	24	CO	CO	37	Better	"Against the seizures I get medication, Orfiril and Keppra. Semi-annual neurological checks. I am hard of hearing and forgetful"
#10	51	CO	CO	66	Better	"It was an emergency intervention after perforation of my sphenoid sinus during paranasal surgery. Since the intervention I have no more double images"
#11	53	CO	CO	60	Better	" I fell from a Hayloft and had a severe head injury. The palsies and the feeling of weakness remained, but since the embolization I can see better. "
#12	51	CO	CO	58	Better	"I was transferred because of a fistula and pulse-synchronous tinnitus after my accident. These complaints have been remedied by the treatment, however I still have a permanent murmuring noise in the left ear and occasionally a whistle in both ears. Sincerely yours“
#13	63	CO	CO	80	Better	-
*#14*	*55*	CO	*CO*	*Deceased in 2010* *at the age of 63*	*-*	*-*
*#15*	*78*	CO	*CO*	*Deceased in 2013* *at the age of 93*	*-*	*-*
#16	41	D	D	52	Better	„The first embolization was successful, but the fistula recurred. The second embolization one year later bought a long lasting success, since then no more fistula associated symptoms occurred.“
#17	42	CO	CO	55	Better	-
#18	36	CO	CO	53	Better	" I still have double images. Sometimes I have headache and feel dizzy, but I am satisfied as it is right now"
*#19*	*62*	*D*	*CO*	*Deceased in 2010* *at the age of 63*	*-*	*-*
		*→ Secondary occlusion →*				
#20	66	CO	CO	76	Better	„My massive, insatiable bleeding after paranasal surgery was stopped by your intervention and never recurred. I live in a retirement home and become best cared by my children. I see my general practitioner for regular checks. Until now no symptoms of a fistula recurred“
#21	65	CO	CO	77	Equal	"One eye is blind, caused by my accident. On the right eye the visual performance is very good. Until now everything is OK"
#22	40	CO	CO	52	Better	„Unfortunately I cannot provide more precise information because I was still in a coma after the accident. All information is based on my husband's memory "
#23	68	D	CO	71	Better	
		*→ Secondary occlusion →*				
#24	39	CO	CO	52	Better	
#25	53	CO	CO	58	Better	„It was a very good operation at night, it saved my life! Thanks to the whole team. Since my accident I sometimes have hallucinations, but in general all is right again. My eyesight is much better and I do a lot of sports. Thank you!"

Abbreviations: LFU = long-term follow-up, CO = Complete occlusion, D = Downgrade

### Patient-reported long-term follow-up

#### Questionnaire response rate & duration

The standardized questionnaire was sent to all included patients. The response rate was 96% (24/25 patients). In one case (1/25, 4%, pat. # 6) long-term follow-up data is missing. This 46-year-old patient, who had successfully and uneventfully been treated for his trauma-related dCCF in one session in 07/2012, had shown no fistula recurrence within a five month imaging follow-up interval, but did not respond to our interview request for unknown causes. Three patients (pat. # 14, 15 & 19) had died in the meantime, among them the two patients in which complications occurred during the intervention (pat. # 14 & 19). The third patient (pat. #15) had died 13 years after successful fistula occlusion at the age of 92 years due to her congestive heart failure. In all of these three cases the questionnaire had been completed by the patients´ relatives together with their general practitioner, however as this is an evaluation of patients’ personal opinion their feedback was not included. The rate of considered feedback is thus 84% (21/25 patients)

Duration of long-term follow-up defined as the median time interval between treatment and date of the interview as indicated on the questionnaire was 143 months (range: 35–226) or 11 years (range: 2–18) respectively. The median patient’s age at the date of the interview was 62 years (range: 37–96).

#### Recovery rates

The recovery rates divided according to the different symptom groups are shown in Table 6. 13/17 patients (76%) initially presenting with chemosis, exophthalmos, retroorbital pain or ophthalmoplegia and 10/17 (58%) with initially diminished visual acuity stated nowadays to be symptom-free in the long-term follow-up interview. Among the patients who stated to have remained constantly symptomatic with regard to these symptoms, there were two patients with trauma-related enucleation of the ocular bulb and the two patients with multiple sclerosis and progressive supranuclear palsy respectively.

**Table 6:** Long-term recovery rates.

ID	Chemosis, Exophthalmos, Retroorbital pain, Ophthalmoplegia		Diminished visual acuity		Pulsatile Tinnitus		Headache		Vertigo	
	*Prior to the intervention*	*Current state*	*Prior to the intervention*	*Current state*	*Prior to the intervention*	*Current state*	*Prior to the intervention*	*Current state*	*Prior to the intervention*	*Current state*
#1	+	+	-	-	+	-	-	-	-	-
#2	-	-	-	-	-	-	-	-	-	-
#3	+	+	+	+	-	-	-	-	-	+
#4	+	+	+	+	+	-	-	-	+	+
#5	+	-	+	-	+	-	+	-	-	-
*#6*	*(not included)*									
#7	-	-	+	-	+	-	+	+	+	-
#8	+	-	+	-	+	-	+	-	+	-
#9	-	-	-	+	+	+	+	-	+	+
#10	+	-	+	-	-	-	+	-	-	-
#11	+	-	+	-	-	-	-	-	-	-
#12	+	-	+	-	+	-	+	+	-	-
#13	+	-	+	-	+	-	-	-	-	-
*#14*	*(not included)*									
*#15*	*(not included)*									
#16	+	-	+	-	+	-	+	-	-	-
#17	+	-	+	-	+	+	+	+	-	-
#18	+	+	+	+	+	+	+	+	+	+
*#19*	*(not included)*									
#20	-	-	-	-	-	-	-	-	-	-
#21	+	-	+	-	-	-	+	-	-	-
#22	+	-	+	+	N/A	-	+	+	+	+
#23	+	-	+	+	-	-	+	-	-	-
#24	+	-	+	+	-	-	N/A	N/A	N/A	N/A
#25	+	-	+	-	-	-	+	-	-	-
	17/21	4/21	17/21	7/21	11/21	3/21	13/21	5/21	6/21	5/21
	Recovery rate: 13/17 (76%)		Recovery rate: 10/17 (58%)		Recovery rate: 8/11 (72%)		Recovery rate: 8/13 (61%)		Recovery rate: 1/6 (17%)	

+ = symptomatic,— = not symptomatic

The recovery rates for the symptom groups “pulsatile tinnitus”, “headache” and “vertigo” were 8/11 (72%), 8/13 (61%) and 1/6 (17%). (Table 6)

Deterioration of pre-existing symptoms was not observed. Except for one patient with new-onset vertigo, none of the initially asymptomatic patients reported on newly developed complaints during the long-term follow-up. (Table 6)

#### Other aspects

One patient initially presented with epileptic seizures, which completely disappeared after successful endovascular fistula occlusion. Three patients developed a convulsive disease in the further course due to their extensive posttraumatic brain lesions. Three patients initially presented with stroke-like neurological deficits due to their fistula related extensive AV-shunt volume and where completely cured. Ten patients initially presented with trauma related intracranial hemorrhage. Except for patient #14 with treatment associated ischemic stroke and secondary bleeding in the infarct territory no recurrent or newly apparent ICB was reported during the long-term follow-up interval.

#### Overall statement on the long-term treatment result

The patient’s subjective benefit from treatment in the long-term follow-up was good. Most of the patients (19/21, 90%) felt they benefited from the treatment („Better“). 2/21 patients (10%) subjectively did not experience any change by the treatment („Equal“). None of the patients remained unsatisfied with the treatment result in the long-term comparison („Worse“). (Table 7)

**Table 7:** Summary statement.

Statement	n	%
Better	19/21	90%
Equal	2/21	10%
Worse	0/21	0%

## Discussion

Endovascular occlusion of dCCF is nowadays performed in centers and has evolved to a well-established treatment for these vascular conditions. There are some well-structured follow-up analyses in literature which report on complication rates and angiographic results of this method. [3,5] However their number is limited and yet there is no publication on how the patients themselves perceive the treatment success.

By the 1980s direct CCFs were preferentially treated with intra-arterial detachable balloon occlusion as first described by Serbinenko. [3,10] For many years most of the author preferred detachable balloon as initial occlusion devices to treat traumatic direct CCF. However, each study reported a certain rate of failure because of being unable to position correctly the balloon within the fistula. [1,3,11–19]

Since detachable balloons were taken off the market some years ago coil embolization and liquid adhesives are now the mainstay of treatment. [11,20–23] Covered stents also may be used in cases with limited vessel tortuosity and are especially useful with large ICA tears. [11]

Liquid embolic agents, such as histoacryl glue (N-butyl-2 cyanoacrylate n-BCA) and Onyx (new liquid polymeric embolic agent) always carry the risk of embolic agent reflux into the ICA during injection. To avoid this, a removable remodelling balloon should be temporarily inflated within the lumen at the fistulous portion of the ICA to close the fistula tract during injection.[3]

Summing up, anticipation of the necessary embolic material before the procedure is really critical to avoid adverse effects or complications. To get a good anticipation of choosing embolic material for the fistula, an insightful understanding of the fistula sizes and hemodynamcis is crucial.[3]

With this article we expand the experience on endovascular fistula therapy around the aspect of the patient reported outcome several years after treatment.

The baseline characteristics of our collective in terms of age and gender distribution go along with others described. [3,5] Analogous to other case series the etiology of our patients’ dCCFs was mostly traumatic, fewly aneurysm related and rarely caused by complicative sphenoidal surgery. [1,2]

Occlusion rate in the last imaging follow up was 96% (24/25 patients). We observed a considerable rate of DOB secondary pressure loss or dislocation leading to early fistula recurrence (62%, 8/13 patients) and could demonstrate, that secondary fistula occlusion in the months following primary endovascular fistula downgrading is a common phenomenon (75%, 3/4 cases). Complication rate was 8% (2/25). All these metrics and observations match with the findings described in literature. [3,5]

Compared to other studies (6 m [4], 17.3 m [5], 20 m [6], 3.8 y [7], 4.4 y [8]), we report on an extensively longer observational period. Duration of our median FU interval was 4 months (range: 0–202 m) for the last imaging FU and 143 months / 11 years (range: 35–226 m / 2–18 y) for the patient reported outcome respectively.

First of all endovascular treatment of a dCCF is a highly effective treatment method leading to an immediate therapy success in the vast majority of cases. All the patients in need of at least one more treatment session either remained symptomatic or showed early recurrence of their initial symptoms within a median time interval of 14 days after the first intervention.

Only one out of 21 patients (pat #17) with eye related symptoms such as diminished visual acuity, chemosis, exophthalmos, retroorbital pain, and ophthalmoplegia reported on a secondary improvement of his complaint within weeks following primary fistula occlusion achieved in one single treatment session, while all other patients showed either a sustainable immediate cure or remained constantly symptomatic. Hence typically eye-related symptoms are the dominant symptoms of a dCCF and show a very good response to endovascular therapy, rather with an “on/off” characteristic than in gradual manner–an experience we share with other authors. [9,24] But also within the symptom groups “pulsatile tinnitus” (8/11, 72%) and “headache” (8/13, 61%) and patients report on a good recovery rates.

Furthermore, this treatment method offers an “immediate feedback”. Inadequate therapy results come either up as an acute complication or as an early fistula recurrence within the first few days after initial treatment (median: 14 days, range: 2–49 days). When this critical phase is over therapy success achieved is very sustainable and, on the contrary, offers the chance of further improvement due to secondary fistula obliteration. Just like in the studies of other authors [3,5] none of our patients worsened or developed new symptoms suggestive of a recurrent fistula during the follow-up period.

Subjective perception of the therapy results on the part of the patients is good. Most of them (19/21, 90%) felt they benefited from the treatment. Even if endovascular supply of a dCCF is mandatory in most of the cases, we consider this fact to be an enriching aspect.

There are several limitations of this study that need to be mentioned. First of all, the small sample size of our retrospective single-center study hampers definite conclusions. Furthermore, we did not perform another objective medically documented current status survey, but only conducted standardized interviews based on the patient´s statements, which may be the most considerable limitation of our work. We excluded the follow-up feedback of the relatives of the three deceased patients, however also the free-text answers of the patients # 2, 4 and 22 suggest that their relatives were involved in answering the questionnaire. In principle, we consider their statements to be representative, but we cannot safely exclude an associated bias.

Additionally clinical data was collected as it was documented in the medical records and does not result from a firmly designed prospective study protocol with standardized clinical examinations at pre-defined time points. This for example also applies to any ophthalmologic examination by an ophthalmologist, which documented in 23/25 cases prior to the intervention, whereas corresponding follow-up control examinations were performed in irregular manner in only a part of the patients. This leaves an amount of uncertainty that a structured medical examination would have yielded a different result from the patient's subjective complaints. On the other hand, exactly the latter parameter was the main focus of our study, which is why we consider the resulting bias to be justifiable.

Summing up our data confirm the experience of other authors concerning the therapy success and complication rates of endovascular treatment of dCCF. In yet another substantially longer follow-up interval patients did not report on symptoms suspicious of delayed fistula recurrence. This therapy option not only offers excellent angiographic results and mostly immediate clinical cure, but also comes up with a long-lasting stable subjective benefit for the patients.

## Supporting information

S1 File Questionnaire. Long-term follow-up interview form provided to all patients.
(DOC)
