# Supplementary material for: Endovascular therapy of direct dural carotid cavernous fistulas – A therapy assessment study including long-term follow-up patient interviews
Source: PLoS One. 2019 Oct 17;14(10):e0223488. doi: 10.1371/journal.pone.0223488 (PMC6797194; doi:10.1371/journal.pone.0223488)
Supplement: S1 File — Long-term follow-up interview form provided to all patients. (DOC) [file pone.0223488.s001.doc]

**Supplement S1 File**

**QUESTIONNAIRE**

*Long-term follow-up after endovascular therapy of intracranial dural arteriovenous fistulas*

| **Did / Do you suffer from …** | **BEFORE**  endovascular treatment | | **IMMEDIATELY AFTER**  endovascular treatment | | **TODAY** | |
| --- | --- | --- | --- | --- | --- | --- |
|  | **YES** | **NO** | **YES** | **NO** | **YES** | **NO** |
| … Chemosis, Exophthalmos, retroorbital pain or Ophthalmoplegia? |  |  |  |  |  |  |
| … a diminished visual acuity? |  |  |  |  |  |  |
| … epileptic seizures? |  |  |  |  |  |  |
| … ischemic stroke ? |  |  |  |  |  |  |
| … intracranial hemorrhage? |  |  |  |  |  |  |
| … headache? |  |  |  |  |  |  |
| … a pulsatile tinnitus? |  |  |  |  |  |  |
| … vertigo? |  |  |  |  |  |  |
| …. cognitive deficits?  (e.g concentration disorders) |  |  |  |  |  |  |
| … any other symptoms, that in your opinion are related to your fistula ?  *(Please describe - Free text)* |  | |  | |  | |

| Please provide a summary statement on the development of your fistula-related symptoms over time by choosing one out of three categories.  Summing up the general course do you feel that your complaints have become …  *(Please mark as appropriate)* | | |
| --- | --- | --- |
| Better | Equal | Worse |
| … after treatment. | | |

| Is there anything else you want to tell us? *(Free text)* |
| --- |
|  |
|  |

THANK YOU FOR YOR PARTICIPATION!
